# Spatial Patterns of Parrotfish Corallivory in the Caribbean: The Importance of Coral Taxa, Density and Size

**DOI:** 10.1371/journal.pone.0029133

**Published:** 2011-12-27

**Authors:** George Roff, Mary H. Ledlie, Juan C. Ortiz, Peter J. Mumby

**Affiliations:** 1 Marine Spatial Ecology Lab, School of Biological Sciences, University of Queensland, St Lucia, Australia; 2 Nature Seychelles, Roche Caiman, Mahé, Seychelles; Swansea University, United Kingdom

## Abstract

The past few decades have seen an increase in the frequency and intensity of disturbance on coral reefs, resulting in shifts in size and composition of coral populations. These changes have lead to a renewed focus on processes that influence demographic rates in corals, such as corallivory. While previous research indicates selective corallivory among coral taxa, the importance of coral size and the density of coral colonies in influencing corallivory are unknown. We surveyed the size, taxonomy and number of bites by parrotfish per colony of corals and the abundance of three main corallivorous parrotfish (*Sparisoma viride*, *Sparisoma aurofrenatum*, *Scarus vetula*) at multiple spatial scales (reefs within islands: 1–100 km, and between islands: >100 km) within the Bahamas Archipelago. We used a linear mixed model to determine the influence of coral taxa, colony size, colony density, and parrotfish abundance on the intensity of corallivory (bites per m^2^ of coral tissue). While the effect of colony density was significant in determining the intensity of corallivory, we found no significant influence of colony size or parrotfish abundance (density, biomass or community structure). Parrotfish bites were most frequently observed on the dominant species of reef building corals (*Montastraea annularis*, *Montastraea faveolata* and *Porites astreoides*), yet our results indicate that when the confounding effects of colony density and size were removed, selective corallivory existed only for the less dominant *Porites porites*. As changes in disturbance regimes result in the decline of dominant frame-work building corals such as *Montastraea* spp., the projected success of *P. porites* on Caribbean reefs through high reproductive output, resistance to disease and rapid growth rates may be attenuated through selective corallivory by parrotfish.

## Introduction

Disturbances on coral reefs affect key demographic processes, directly resulting in changes in population size [1] and composition [2] of coral communities. On Caribbean reefs, a systematic decline in the abundance of long-lived framework building corals [3], [4] has resulted in shift in dominance towards short-lived “weedy” coral species [5]. Such dramatic shifts in ecosystem structure and function have led to a renewed interest in processes that influence demographic rates in corals [6]. One such process is corallivory [7], which, at least in post-recruitment sized corals, largely manifests as a source of chronic partial mortality [8].

In the Caribbean region, parrotfish from the genus *Sparisoma* and *Scarus* [7], [9] are largely responsible for corallivory. The extent of parrotfish corallivory varies considerably [7], and accounts between 0 - 4% of total live corals [8], [10], [11], [12] and up to 9% of all bites by adult parrotfish (*Sparisoma viride* [10]). Previous studies [13], [14], [15] have identified two forms of corallivory by parrotfish; ‘spot biting’ (where parrotfish excavate individual bites distributed across the entire skeleton), and ‘focused biting’ (where repeated overlapping parrotfish bites result in extensive removal of large patches of coral tissue).

To interpret the likely demographic consequences of corallivory on coral communities, it is critical to have a clear understanding of the influence of coral species, size and density on patterns of corallivory. The intensity of corallivory (as measured by number of bite scars per colony or per area, [7]) varies considerably among reef habitats [9], [11], [15], and in some instances may lead to local exclusion of coral taxa [16], [17]. To date, most studies have observed a selective preference for the most dominant corals, namely *Montastraea annularis* and *Montastraea faveolata* [9], [13], [18], [19]. Local reports also indicate that *Porites* spp. are also commonly grazed [7], [12], [16], [17], with particularly high rates of corallivory reported on *Porites astreoides* [15]. However, the explicit importance of coral size and the density of coral colonies in influencing the intensity of corallivory have not yet been investigated.

**Figure 1:**
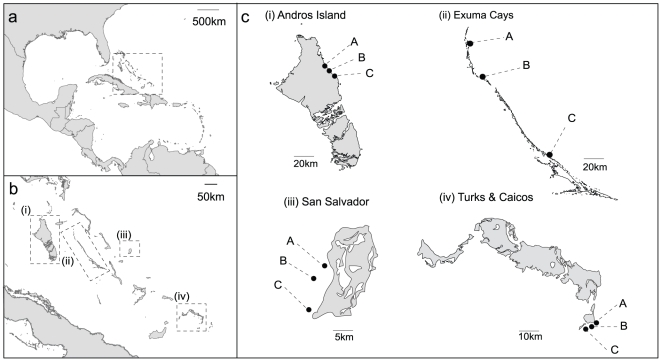
Map of study locations. Caribbean region (a), Bahamas (b) and inset (c) of study islands (i. Andros Island, ii. Exuma Cays, iii. San Salvador, iv. Turks & Caicos).

Here we conduct spatial surveys of corallivory to investigate the effects of coral species, size and density on the intensity of corallivory. We used a regional data set collected from fore-reef environments in the Bahamas archipelago between 2002 and 2004. Following a hierarchical sampling design, we surveyed three reefs nested within four islands (Andros Island, Exuma Cays, San Salvador Island, Turks & Caicos, Figure 1), representing multiple spatial scales (tens to hundreds of kilometers). We quantified the intensity of corallivory (number of bites per m^2^ of coral tissue), coral parameters (density, taxa and size) and parrotfish abundance (density and biomass of three main parrotfish species, *Sparisoma aurofrenatum*, *Sparisoma viride* and *Scarus vetula*). Specifically, we aim to determine: 1) patterns of selective corallivory by parrotfish on coral taxa, 2) the relationship between coral colony density and size on the intensity of corallivory, 3) the relationship between parrotfish abundance (density, biomass and community structure) on the intensity of corallivory, 4) patterns of corallivory across multiple spatial scales.

## Results and Discussion

### Structure of the corallivore assemblage

**Figure 2:**
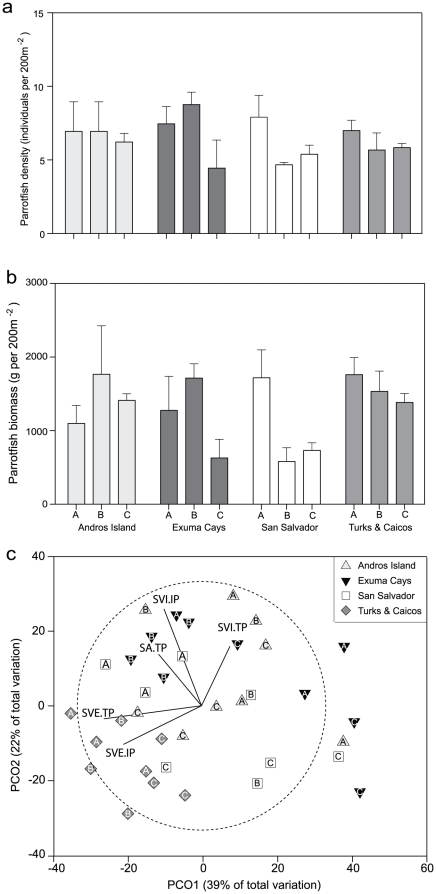
Parrotfish dynamics. Parrotfish density (a) and biomass (b) between islands and reefs (±SE), and Principle Components ordination (PCO) if parrotfish biomass community structure (c). SVI = *Sparisoma viride*, SVE = *Scarus vetula*, SA = *Sparisoma aurofrenatum*, TP = terminal phase, IP = initial phase). Error bars represent ± SE.

Our results indicate that total parrotfish density was variable across Islands throughout Bahamas archipelago, varying from 4.5±1.9 individuals per 120 m^2^ at San Salvador to 8.8±0.8 individuals per 120 m^2^ in the Exuma Cays (Figure 2a). Parrotfish biomass was more variable than density, varying from 586.7±186 g per 120 m^2^ (± SE) at San Salvador to 1767.4±658 g per 120 m^2^ in the Exuma Cays (Figure 2b). These results are similar to those reported elsewhere in the Caribbean (e.g. Belize [12]), yet are considerably higher than heavily fished regions (e.g. Jamaica [20]). A principle coordinates ordination analysis (PCO, [21]) of parrotfish community structure indicated that initial and terminal phases of *Scarus vetula* dominated parrotfish communites in the Turks & Caicos Islands, whilst the remaining three islands showed no clear clustering and the ordination largely explained by the initial and terminal phases of *Sparisoma viride*, and the terminal phase of *Sparisoma aurofrenatum* (Fig. 2c).

### Structure of the coral assemblage

**Figure 3:**
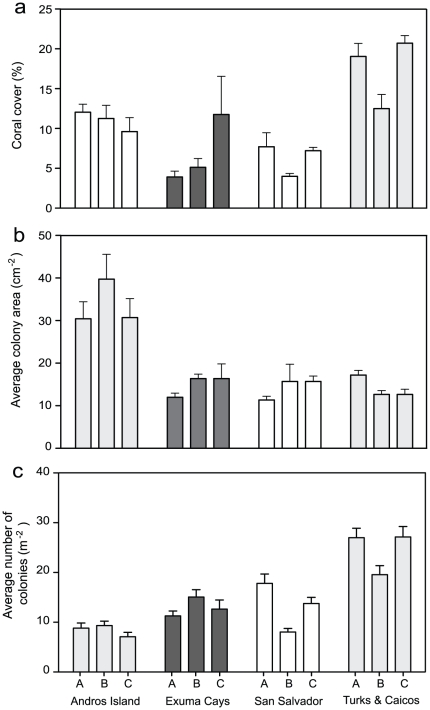
Coral community structure and corallivory. a) Coral cover, b) colony area, and c) colony density across islands and reefs (c). Error bars represent ± SE.

Coral cover throughout the Bahamas is generally low [22], [23], [24] following the severity of the 1998 coral bleaching event [25] and hurricane Frances in 2004. Coral cover averaged 10.4±1.0% across all sites, and ranged from 2.5% to 22.5% (Figure 3a). We identified a total of 15 coral taxa (AGAR = *Agaricia* spp., MFAV = *Montastraea faveolata*, MANN = *Montastraea annularis*, MCAV = *Montastraea cavernosa*, PPOR = *Porites porites*, PAST = *Porites astreoides*, SSID = *Siderastrea siderea*, EUSM = *Eusmilia fastigiata*, FFRA = *Favia fragum*, MDEC *Madracis decactis*, STEP = *Stephanocoenia* spp., DIPL = *Diploria labyrinthiformis*, DICH = *Dichocoenia* spp., MANI = *Manicina* spp., MYCE = *Mycetophyllia* spp, Table 1). The average colony size recorded across all sites was relatively consistent across reefs (Figure 3b) with the exception of Andros Island, where colonies were larger on average (39.7±5.8 cm^2^). The average colony density varied three to four fold across reefs (Fig. 3c).

**Table 1:** Percent cover of coral taxa.

Taxa	Andros Island			Exuma Cays			San Salvador			Turks & Caicos Island		
	a	b	c	a	b	c	a	b	c	a	b	c
AGAR	0.12±0.1	0.17±0.1	0.12±0.1	-	0.16±0.1	0.27±0.1	0.51±0.1	0.05±0.1	0.15±0.1	0.63±0.1	0.31±0.1	0.36±0.1
DICH	-	0.01±0.1	-	0.02±0.1	0.01±0.1	0.02±0.1	-	-	0.01±0.1	0.01±0.1	-	-
DIPL	0.05±0.1	0.19±0.1	0.14±0.1	0.03±0.1	0.10±0.1	0.16±0.1	0.03±0.1	0.05±0.1	0.18±0.1	0.08±0.1	0.09±0.1	0.03±0.1
EUSM	-	-	-	0.01±0.1	0.02±0.1	-	0.01±0.1	-	-	0.01±0.1	-	-
FRAG	-	-	-	0.01±0.1	0.01±0.1	-	0.01±0.1	-	-	0.01±0.1	0.02±0.1	0.02±0.1
MADD	-	-	-	-	0.01±0.1	-	-	-	-	0.01±0.1	0.01±0.1	0.02±0.1
MANI	-	-	-	0.01±0.1	0.01±0.1	-	-	-	-	-	-	-
MANN	1.07±0.3	1.76±0.3	0.69±0.1	0.14±0.1	0.41±0.2	1.15±0.1	0.33±0.1	0.14±0.1	0.58±0.1	2.67±0.4	0.88±0.3	3.34±0.4
MCAV	0.33±0.3	0.03±0.1	0.03±0.1	0.10±0.1	0.09±0.1	0.19±0.1	0.09±0.1	0.30±0.1	0.13±0.1	0.04±0.1	0.31±0.1	0.06±0.1
MFAV	0.62±0.2	1.22±0.5	0.62±0.1	0.07±0.1	0.32±0.1	1.59±0.1	0.01±0.1	0.27±0.1	0.28±0.1	0.26±0.1	0.24±0.1	0.51±0.3
MYCE	0.02±0.1	0.01±0.1	0.01±0.1	-	0.01±0.1	0.01±0.1	0.01±0.1	-	0.01±0.1	-	-	0.01±0.1
PAST	0.12±0.1	0.17±0.1	0.28±0.1	0.35±0.1	0.74±0.1	0.58±0.1	0.08±0.1	0.02±0.1	0.03±0.1	0.17±0.1	0.16±0.1	0.16±0.1
PPOR	0.07±0.1	0.10±0.1	0.27±0.1	0.09±0.1	0.30±0.1	0.18±0.1	0.11±0.1	-	0.02±0.1	0.43±0.2	0.08±0.1	0.30±0.2
SSID	0.28±0.1	0.04±0.1	0.02±0.1	0.51±0.1	0.28±0.1	0.22±0.1	0.35±0.1	0.41±0.1	0.20±0.1	0.25±0.1	0.38±0.1	0.45±0.2
STEP	-	-	-	0.03±0.1	-	0.01±0.1	0.03±0.1	-	-	0.07±0.1	0.01±0.1	0.02±0.1

(AGAR = *Agaricia* spp., MFAV = *Montastraea faveolata*, MANN = *Montastraea annularis*, MCAV = *Montastraea cavernosa*, PPOR = *Porites porites*, PAST = *Porites astreoides*, SSID = *Siderastrea siderea*, EUSM = *Eusmilia fastigiata*, FFAV = *Favia fragum*, MDEC *Madracis decactis*, STEP = *Stephanocoenia* spp., DIPL = *Diploastrea labyrinthica*, DICH = *Dichocoenia* spp., MANI = *Manicina* spp., MYCE = *Mycetophyllia* spp.).

### Intensity of corallivory

**Figure 4:**
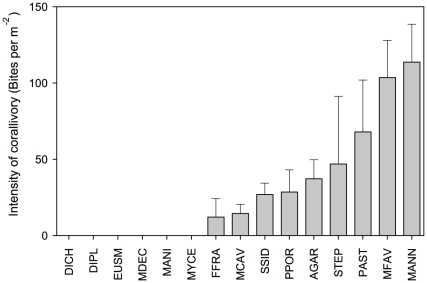
Intensity of corallivory among coral taxa. Average number of bites per m^2^ across coral taxa. Error bars represent ± SE.

We quantified the intensity of corallivory by counting the number of paired parrotfish bite scars [7] within colonies. Only two taxa were consistently bitten across all islands (MANN & MFAV), whilst six taxa were entirely unaffected by corallivory (DIPL, DICH, EUSM, MADD, MANI, MYCE). When standardized for colony area (per m^2^), the most common coral taxa (MANN, MFAV and PAST, Table 1) showed the highest intensity of corallivory (Figure 4), consistent with previous studies of corallivory where MFAV (104±24 bites per m^2^) and MANN (114±25 bites per m^2^) show the greatest prevalence of bite scars [8], [26]. Our results suggest that the intensity of corallivory is generally low throughout the Bahamas archipelago when compared to other habitats [12] and regions of the Caribbean [27], yet the intensity of corallivory within taxa was high for some species within sites (e.g. up to 1068 bites per m^2^ for STEP at Turks & Caicos).

### Patterns of corallivory: importance of coral species, density and size

We used a linear mixed model to determine the effect of coral taxa on the intensity of corallivory (bites per m^2^ of coral tissue), while accounting for variability in colony size, colony density, total coral cover and parrotfish abundance between sites. Total coral cover, colony size, parrotfish biomass and parrotfish community structure were non-significant (p value higher than .25 and the proportion of variability explained lower than 5%) and were excluded from the final model (Table 2). The model found a positive effect of colony density (*β* = 0.0041), taxa and island on the number of bites per m^2^ (Table 2) Taxa explained the largest component of variance (14.3%), while island and colony density explained 12.3% and 5% respectively. The interaction between density and taxa was not significant, indicating that colony density was consistent among taxa (Table 2). Colony size and total coral cover did not have a consistent effect on the overall intensity of corallivory nor among taxa (Table 2). The interaction between colony size and density was not significant, further suggesting that the intensity of corallivory was not dependent upon coral cover.

**Table 2:** PERMANOVA results for intensity of corallivory (bites per dm^2^).

Source	d.f.	SS	MS	ECV	Pseudo-F	P
Colony density	1	6.273	6.273	5	4.64	*
Parrotfish density	1	4.275	4.275	3.9	3.132	ns
Taxa	14	55.684	3.978	14.3	3.671	***
Island	3	23.364	7.788	12.3	7.623	***
Colony density*Taxa	14	0.145	0.145	0	0.142	ns
Colony density*Island	3	7.304	2.435	7.4	2.383	ns
Island*Taxa	40	51.784	1.295	9.5	1.267	ns
Residuals	368	375.97	1.022		47.6	
Total	431	524.8				

(d.f. = degrees of freedom, SS = sum of squares (type I), MS = mean sum of squares, ECV = percent estimated components of variation).

When properly controlled for differences in colony size and density, the intensity of corallivory (bites per m^2^) can be interpreted as a measure of preference. When a significant difference is found between the intensity of corallivory of two coral taxa in pairwise tests, the species with higher bites per m^2^ (when available in equivalent amounts) is selected over the other species. Post-hoc comparisons among the fifteen coral taxa revealed only 10 out of 81 possible pairwise comparisons between taxa were significant (Table 3). In all significant comparisons, one of the pairwise taxa was not bitten by parrotfish, indicating a clear preference for the alternative taxa (Table 3). PPOR was the only taxa with more than two significant interactions (6 out of 14 possible pairwise interactions), indicating a weak but consistent preference for PPOR. Despite significant differences in the intensity of corallivory among islands, no interaction with taxa was observed (Table 2), indicating that preferential corallivory of PPOR was consistent among all four islands. Correlative studies have previously suggested that parrotfish abundance is important in determining the intensity of corallivory within certain coral taxa [12]. Yet, in our model, parrotfish density was marginally significant (*p* = 0.06), the component of variance was minor and lower than other significant variables (3.9%), and no interactions were observed with taxa.

**Table 3:** Pairwise results from PERMANOVA for intensity of corallivory (bites per dm^2^).

	AGAR	DICH^0^	DIPL^0^	EUSM^0^	FFRA	MDEC^0^	MANI^0^	MANN	MCAV	MFAV	MYCE^0^	PAST	PPOR	SSID	STEP
AGAR	-	-	-	-	-	-	-	-	-	-	-	-	-	-	-
DICH^0^	ns	-	-	-	-	-	-	-	-	-	-	-	-	-	-
DIPL^0^	ns	x	-	-	-	-	-	-	-	-	-	-	-	-	-
EUSM^0^	ns	x	x	-	-	-	-	-	-	-	-	-	-	-	-
FFRA	ns	ns	ns	ns	-	-	-	-	-	-	-	-	-	-	-
MDEC^0^	ns	x	x	x	ns	-	-	-	-	-	-	-	-	-	-
MANI^0^	ns	x	x	x	*	x	-	-	-	-	-	-	-	-	-
MANN	ns	ns	ns	ns	ns	*	ns	-	-	-	-	-	-	-	-
MCAV	ns	ns	ns	ns	ns	ns	ns	ns	-	-	-	-	-	-	-
MFAV	ns	ns	ns	ns	ns	ns	ns	ns	ns	-	-	-	-	-	-
MYCE^0^	ns	x	x	x	ns	x	x	ns	ns	ns	-	-	-	-	-
PAST	ns	ns	ns	ns	ns	ns	ns	ns	ns	ns	ns	-	-	-	-
PPOR	ns	*	*	*	ns	*	***	ns	ns	ns	*	ns	-	-	-
SSID	ns	ns	ns	ns	ns	ns	ns	ns	ns	ns	ns	ns	ns	-	-
STEP	ns	ns	ns	ns	ns	ns	w	ns	ns	ns	*	ns	ns	ns	-

(x = not consistent between islands, ns = not significant, * = p<0.05, ** = p<0.01, *** = p<0.001). Taxa marked with ^0^ were present across all sites but not bitten by parrotfish.

Consistent with previous studies, we found that the intensity of corallivory was highest on the most common framework building taxa (MANN, MFAV and PAST, Figure 4). The fact that the intensity of corallivory in these taxa occurs in direct relation to their abundance highlights the potential importance of corallivory on Caribbean reefs. Yet, when accounting for coral size and density, and parrotfish abundance (density, biomass and community structure), we found only evidence for a weak, but significant preference by parrotfish for *Porites porites* (PPOR, Table 3). These results are consistent with research on corallivory from the Florida Keys and Belize showing a preference of PPOR grazing by parrotfish [16], [17], [27]. At similar levels of parrotfish biomass, intense corallivory by parrotfish was considered a key factor in the exclusion of PPOR in Belizean backreef environments, resulting in coral communities dominated by the less palatable PAST [16]. Further experiments excluding parrotfish at these sites using caging studies resulted in 6 fold increases in PPOR growth rates after a 24 month period [16]. Preferential grazing of PPOR over PAST is attributed to differences in growth form and subsurface corallum hardness [16]; PPOR represents a ‘softer’ branching growth form, whereas PAST exhibited a harder skeleton under mechanical testing and is mound shaped, resulting in minimal coral mortality even under higher grazing pressures (even when PAST is observed being grazed by parrotfish, [16]).

Our results reinforce earlier studies suggesting that morphology may play a key role in determining the grazing behaviour of parrotfish [9], [16]. Parrotfish are commonly observed biting lobes or rims of massive corals [14], and adult *S.viride* show strong preference in biting convex surfaces (such as PPOR) over flat surfaces (such as PAST) [9]. Of all taxa consumed in the present study, PPOR is a representative branching growth form, commonly referred to as the ‘finger coral’ due to skeletal protusions. The other grazed taxa (MANN, MFAV, MCAV, PAST, STEP & FFRA) have sub-massive to massive growth forms, providing reduced three dimensional structure for parrotfish to bite, although bites are frequently taken on the most convex parts of the colony surface. While AGAR is perhaps the exception to this rule, with a complex morphology (encrusting to foliaceous growth form), it has a higher skeletal density than massive growth forms [28], which may be critical in determining the intensity of corallivory by parrotfish [16]. Despite the clear selective preference for PPOR, our results indicate no selective preference exists for another branching coral (MDEC), suggesting that morphology may not be the sole factor in determining patterns of selective corallivory, and that other underlying factors may account for parrotfish grazing on PPOR.

### Corallivory electivity

**Figure 5:**
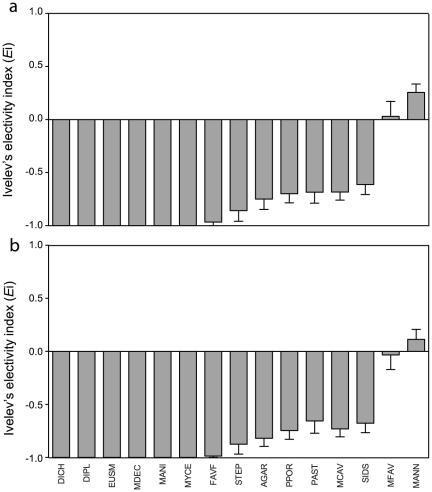
Electivity by parrotfish for coral taxa based upon a) colony-based electivity, and b) area based electivity. Electivity was calculated following Ivlev's electivity index (*E*_i_), where positive values indicate a preference by parrotfish, and negative values indicate avoidance by parrotfish. Error bars represent ± SE.

Previous studies have used electivity indices to measure selective corallivory among taxa [12], [27], [29], [30], [31], [32]. To allow comparisons with previous studies (e.g. [12]), we determined selectivity for different coral species using Ivlev's electivity index (*E*_i,_) according to colony-based and area-based selectivity metrics [27]. The results of both metrics indicate that PPOR was avoided by parrotfish (Figure 5a,b), which is consistent with the intensity of corallivory (Figure 4). Moreover, MANN and MFAV were found to be preferred substrata, as has been found in previous studies that used electivity (e.g. [12]). To compare the electivity results with our previous analysis of the intensity of corallivory, we ran a linear model with electivity as the response variable and colony density, average colony size, and taxa as the predictors. For both colony-based (Table 4) and area based electivity (Table 5), interactions between the covariates and the categorical predictors were highly significant. The model found colony size within taxa to be an important correlate of electivity for both colony-based (AGAR *β* = −0.019, STEP *β* = 0.007, Table 4) and area-based electivity (PAST *β* = 0.015, STEP *β* = 0.007, Table 5), thus confirming our concern that measures of electivity can be confounded by covariates (in this instance interspecific differences in colony size). These interactions indicate a violation of the assumption of equality of slopes (one of the main assumptions of our model, [33]), implying that it was not possible for the model to appropriately remove the effect of the covariates on the from the effect of categorical predictors [33].

**Table 4:** PERMANOVA results for colony-based E_i_ (Ivlev's electivity index).

Source	d.f.	SS	MS	ECV	Pseudo-F	*p*
Colony density	1	20.662	20.662	13.4	89.174	***
Average colony size	1	3.509	3.509	5.4	16.835	***
Taxa	14	37.741	2.696	18.7	14.798	***
Island	3	4.319	1.440	7.1	12.847	**
Reef*Island	8	0.878	0.110	0.0	0.643	ns
Colony density*Taxa	14	3.652	0.261	6.1	1.529	ns
Colony density*Island	3	0.068	0.023	0.0	0.133	ns
Colony density*Reef*Island	8	1.683	0.210	2.4	1.233	ns
Average colony size*Taxa	14	4.675	0.334	6.5	1.957	*
Average colony size*Island	3	3.462	1.154	7.8	6.764	***
Average colony size*Reef*Island	8	3.165	0.396	7.1	2.319	*
Res	351	59.888	0.171	25.4		
Total	428	143.7				

(d.f. = degrees of freedom, SS = sum of squares (type I), MS = mean sum of squares, ECV = percent estimated components of variation).

**Table 5:** PERMANOVA results for area-based E_i_ (Ivlev's electivity index).

Source	d.f.	SS	MS	ECV	Pseudo-F	*p*
Colony density	1	15.8	15.8	10.2	76.273	***
Average colony size	1	4.096	4.096	2.6	21.863	***
Taxa	14	2.288	2.288	22.0	13.664	***
Island	3	1.148	1.148	3.0	14.839	***
Reef*Island	8	0.593	0.071	0.0	0.468	ns
Colony density*Taxa	14	3.480	0.249	2.8	1.568	ns
Colony density*Island	3	0.159	0.053	0	0.335	ns
Colony density*Reef*Island	8	1.361	0.170	0.1	1.073	ns
Average colony size*Taxa	14	6.018	0.430	5.3	2.711	**
Average colony size*Island	3	3.913	1.304	5.2	8.225	***
Average colony size*Reef*Island	8	3.241	0.405	4.1	2.555	*
Res	351	55.657	0.159	44.7		
Total	428	129.8				

(d.f. = degrees of freedom, SS = sum of squares (type I), MS = mean sum of squares, ECV = percent estimated components of variation).

In contrast to the pairwise results from the intensity of corallivory, where PPOR had the highest number of significant comparisons (6 out of 14 possible pairwise interactions), MANN and MFAV had the highest number of significant comparisons for both metrics of *E*_i_ (13 out of 14 possible pairwise interactions, Table 6 & 7). Unlike the models for both metrics of *E*_i_, interactions between the covariates and categorical predictors were not significant for the intensity of corallivory (Table 3). This indicates that the model was successful in removing the confounding effect of covariates, and strongly implies that the selective corallivory for PPOR is independent of the other factors studied. Given that the two models indicate contrasting patterns of selective corallivory, and only the intensity of grazing model was successful in removing the effect of the covariates, we conclude that the preference for PPOR is independent of colony density, size, and location, while the preference for MFAV and MANN is an artifact of their abundance and size. Regardless of the metric used (any electivity index or intensity of corallivory), our results highlight that not fully incorporating both the structure in the dataset and the potential effect of covariates can result in misleading results in determining patterns of electivity.

**Table 6:** Pairwise results from PERMANOVA for colony-based E_i_ (Ivlev's electivity index).

	AGAR	DICH^0^	DIPL^0^	EUSM^0^	FFRA	MDEC^0^	MANI^0^	MANN	MCAV	MFAV	MYCE^0^	PAST	PPOR	SSID	STEP
AGAR	-	-	-	-	-	-	-	-	-	-	-	-	-	-	-
DICH^0^	ns	-	-	-	-	-	-	-	-	-	-	-	-	-	-
DIPL^0^	ns	x	-	-	-	-	-	-	-	-	-	-	-	-	-
EUSM^0^	*	x	x	-	-	-	-	-	-	-	-	-	-	-	-
FFRA	*	ns	ns	ns	-	-	-	-	-	-	-	-	-	-	-
MDEC^0^	*	x	x	x	ns	-	-	-	-	-	-	-	-	-	-
MANI^0^	ns	x	x	x	ns	x	-	-	-	-	-	-	-	-	-
MANN	***	***	***	***	***	***	***	-	-	-	-	-	-	-	-
MCAV	ns	ns	ns	ns	ns	ns	ns	***	-	-	-	-	-	-	-
MFAV	***	***	***	***	***	**	*	ns	**	-	-	-	-	-	-
MYCE^0^	*	x	x	x	x	x	x	***	x	***	-	-	-	-	-
PAST	ns	ns	ns	ns	ns	ns	ns	***	ns	***	ns	-	-	-	-
PPOR	ns	ns	ns	*	ns	ns	ns	***	ns	**	ns	ns	-	-	-
SSID	ns	ns	ns	ns	ns	ns	ns	***	ns	***	ns	ns	ns	-	-
STEP	ns	ns	*	ns	ns	ns	ns	***	ns	**	ns	ns	ns	ns	-

(x = not consistent between islands, ns = not significant, * = p<0.05, ** = p<0.01, *** = p<0.001). Taxa marked with ^0^ were present across all sites but not bitten by parrotfish.

**Table 7:** Pairwise results from PERMANOVA for area-based E_i_ (Ivlev's electivity index).

	AGAR	DICH^0^	DIPL^0^	EUSM^0^	FFRA	MDEC^0^	MANI^0^	MANN	MCAV	MFAV	MYCE^0^	PAST	PPOR	SSID	STEP
AGAR	-	-	-	-	-	-	-	-	-	-	-	-	-	-	-
DICH^0^	ns	-	-	-	-	-	-	-	-	-	-	-	-	-	-
DIPL^0^	ns	x	-	-	-	-	-	-	-	-	-	-	-	-	-
EUSM^0^	*	x	x	-	-	-	-	-	-	-	-	-	-	-	-
FFRA	*	ns	ns	ns	-	-	-	-	-	-	-	-	-	-	-
MDEC^0^	ns	x	x	x	ns	-	-	-	-	-	-	-	-	-	-
MANI^0^	ns	x	x	x	ns	x	-	-	-	-	-	-	-	-	-
MANN	***	***	***	***	***	***	**	-	-	-	-	-	-	-	-
MCAV	ns	ns	ns	ns	ns	ns	ns	***	-	-	-	-	-	-	-
MFAV	***	***	***	**	***	***	*	ns	**	-	-	-	-	-	-
MYCE^0^	ns	x	x	x	x	x	x	***	x	**	-	-	-	-	-
PAST	ns	ns	ns	ns	ns	ns	ns	***	ns	***	ns	-	-	-	-
PPOR	ns	ns	ns	ns	*	ns	ns	***	ns	***	ns	ns	-	-	-
SSID	ns	ns	ns	ns	ns	ns	ns	***	ns	***	ns	ns	ns	-	-
STEP	ns	ns	**	ns	ns	ns	ns	***	ns	*	ns	ns	ns	ns	-

(x = not consistent between islands, ns = not significant, * = p<0.05, ** = p<0.01, *** = p<0.001). Taxa marked with ^0^ were present across all sites but not bitten by parrotfish.

### Potential difficulties in interpreting records of corallivory from bite scars

While our results indicate a weak selection preference for PPOR over other coral taxa, we must consider potential shortcomings of the metric of corallivory (intensity of corallivory, bites per m^2^). Although surveys of bite scars are a common metric of corallivory (sensu [7]), the approach implicitly assumes that patterns in bite density reflect patterns in the rate at which parrotfish feed on corals. However, the persistence of a bite scar is dependent upon the regenerative capacity of the coral colony, which can vary significantly among taxa [34], [35] and potentially among habitats or locations [36], [37]. In theory, if a coral is much slower at repairing lesions, it will tend to have a higher density of bite scars over time even if the actual incidence of predation is identical to that of other corals. Conversely, if a coral heals more rapidly, it will tend to show a lower density of bite scars. Considering that growth rates of PPOR are up to 5 fold that of MANN [38], our results may actively underestimate the intensity of parrotfish corallivory on PPOR due to its inherent high regenerative capacity and subsequent rapid lesion healing. If this is the case, it implies that we have merely underestimated the preference for PPOR and our overall conclusions are unaffected.

### The importance of parrotfish abundance

Our results imply that parrotfish density is not a strong driver of corallivory on Bahamian reefs. This seems surprising, given that previous studies have indicated correlations do exist between the intensity of corallivory on certain coral taxa and parrotfish species (e.g. MFAV and *Sp. aurofrenatum* densities, [16]). The lack of pattern in our study is unlikely to be caused by a lack of variance in parrotfish abundance among sites, as we observed variability in density and biomass among islands (Figure 2). However, we expect that a stronger effect of parrotfish abundance would be observed in areas where more intense fishing has created an even stronger gradient in parrotfish abundance and size, such as Jamaica [20].

### Patterns of corallivory at multiple spatial scales

To date, most studies of corallivory from the Caribbean have been conducted at single locations [9], [11], [16], or involved comparisons among habitats on the same reef [12]. Given that we found variation in parrotfish and benthic community structure among reefs (Figure 2), it was surprising to find little variation in corallivory at reef scales (1–100 km) (Table 3). We found no significant interactions between island and any of the other variables in our analysis (Table 3), suggesting that inter-island variability in the intensity of corallivory is not driven by differences in coral cover or parrotfish abundance. Much greater variation occurred at the scale of islands (>100 km), implying that future surveys of corallivory should consider stratification at these larger scales.

### Implications for future reef trajectories

Our results indicate a weak selective preference of PPOR over the other 8 taxa affected throughout the Bahamas archipelago. The response of parrotfish to changes in the availability of coral such as those predicted under future climate change scenarios is unclear [39]. Through rapid growth rates [38], [40] and high reproductive output [41], [42], PPOR is generally considered to be a pioneer species [43] and a ‘winner’ under future climate change scenarios, to the extent that it has already replaced once historically dominant *Acropora* communities at some sites in the Bahamas [44]. Although strongly susceptible to hurricane damage [45], PPOR is generally resistant to both disease [46], [47], [48], and macroalgal overgrowth [17], yet is susceptible to bleaching [49] (but see [50], [51]). However, given the selective preference of the intensity of corallivory on PPOR reported under low rates of corallivory in the present study, and the near exclusion of PPOR under heavy rates of parrotfish grazing reported elsewhere in the Caribbean [16], [17], our results suggest that increases in PPOR dominance may be constrained – though not necessarily prevented – by corallivory. Further, as climate change drives coral reefs towards novel assemblages with low coral cover and higher densities of small colonies [2], [52], critical questions remain as to whether the intensity of corallivory will intensify. Our analysis found higher intensity of corallivory occurred at sites with higher colony densities, which might imply that overall levels of corallivory will increase if reefs become increasingly dominated by higher densities of smaller colonies.

## Methods

The study was conducted between 2002 and 2004 across four islands in the Bahamas region (Andros Island, Exuma Cays, San Salvador Island, Turks & Caicos, Figure 1) under a permit from the Department of Marine Resources as part of the NSF Biocomplexity project. Sampling followed a hierarchical stratified random sampling design, where islands were selected at random, and three reefs (A–C) chosen at random within each island. At each reef, 2–4 sites were selected at random (islands>reef>site). Sampling was restricted to fore-reef environments at all sites (‘Montastraea habitat’, [53]) at a depth of 10 m. To quantify parrotfish dynamics at each site, the biomass and density of initial and terminal phases of the stoplight (*Sparisoma viride*), redband (*Sparisoma aurofrenatum*) and queen parrotfish (*Scarus vetula*) were quantified across ten 30×4 m belt transects per site at 10 m depth. The lengths of individual parrotfish were converted to biomass based on allometric scaling relationships [54]. Principle Coordinates Ordination (PCO, [21]) using Bray-Curtis similarity was used to visualize parrotfish community structure between sites.

To determine coral community structure and the intensity of corallivory per colony, fourteen 1 m^2^ quadrats (divided into 20 cm by 20 cm squares) were haphazardly placed at each site and filmed using a high definition digital video camera (Sony DCR-PC120). Video footage was projected onto a large monitor and individual colonies identified to the lowest possible taxonomic resolution. Colonies were defined as individual ramets (autonomous patches of living coral tissue). The numbers of parrotfish bites on each colony were counted, where bites were identified as paired white or green areas of excavated skeleton where live tissue had been removed. Counting parrotfish bites from video transects may underestimate bite densities when compared with in situ field surveys (e.g. [27]). The areal coverage of each individual coral colony was calculated using Vidana (http://www.marinespatialecologylab.org/resources/vidana/), and the number of bites per m2 (intensity of corallivory, previously termed ‘grazing extent’, [12]) calculated for each taxa.

For all the statistical analysis, site was considered the lower level of replication. A single value for each site was calculated (average or sum depending on the variable) for both categorical and continuous predictors. We used a linear mixed model using permutations with a Type I (sequential) sum of squares to calculate the p values using PERMANOVA [55], where bites per m^2^ was a response variable, parrotfish density, parrotfish biomass, parrotfish community structure (first axis of PCO), total coral cover, coral density, and mean size were covariates, coral taxa was a fixed factor and island and reef nested within island were considered random factors.

By using a type I sum of squares, the model calculates the significance of each of the factors by subtracting the effect of the covariates, allowing us to test the effect of taxa & island independently of the covariates. When a factor (main effect or interactions) in the model was not significant, the p value was higher than .25 and the proportion of variability explained by the factor lower than 5% we removed the factor from the analysis, and the model re-run without the excluded factors following [33].

To allow comparisons with previous studies of parrotfish corallivory (e.g. [12]), we determined selectivity for different coral species by parrotfish using Ivlev's electivity index (*E_i_*, [56]) as:
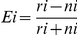
We calculated *E_i_* using colony-based electivity (i.e. where *ri* is the proportion of all parrotfish bites that were taken on the *i*th coral species, and *n_i_* is the proportional abundance based on colony abundance) and for area-based electivity (i.e. where *ri* is the proportion of all parrotfish bites that were taken on the *i*th coral species, and *n_i_* is the proportional abundance based colony area). To determine whether both of these approaches are confounded by colony size and colony density, we repeated the same statistical approach using the linear mixed model as above with E_i_ as the response variable.
